# Inorganic Acid Resistance Performances of Magnesium Phosphate Cement: A Two-Year Observation

**DOI:** 10.3390/ma17225644

**Published:** 2024-11-19

**Authors:** Huaqiang Sun, Kanghong Zhuo, Tianzhu Lin, Shusen Zhuang, Sixiang Kang, Congcong Ma, Lingxi Deng

**Affiliations:** 1 School of Advanced Manufacturing, Fuzhou University, Quanzhou 362251, China; sunhq@fzu.edu.cn (H.S.); zhuokanghong@163.com (K.Z.); tianzhu999@gmail.com (T.L.); zss@fzu.edu.cn (S.Z.); kangsixiang66@gmail.com (S.K.); 2 College of Materials Science and Engineering, Chongqing University, Chongqing 400044, China; congcong.ma@cqu.edu.cn; 3 Chongqing Construction Science Research Institute Co., Ltd., Chongqing 400042, China

**Keywords:** magnesium phosphate cement, inorganic acid-resistance, microstructure, hydration, macro performance

## Abstract

Magnesium phosphate cement (MPC), a cementitious material that hardens through an acid–base reaction, is theoretically expected to exhibit strong acid resistance. However, studies on the durability of MPC in acidic environments remain limited. This study aims to systematically evaluate the acid resistance of MPC in common inorganic acid solutions across various pH levels. By measuring changes in compressive strength, mass loss, apparent changes, pH changes, and the microstructure evolution of MPC under acidic conditions, the mechanisms and influencing factors of its acid resistance are revealed. The results indicate that at pH levels of 1.0 and 2.0, MPC’s resistance to H_2_SO_4_ and HCl erosion is markedly superior compared to its performance against H_3_PO_4_, as evidenced by compressive strength retention, mass loss, and visible erosion. At pH levels above 2.0, MPC demonstrates robust resistance to all tested corrosive media, with compressive strength retention ranging from 68.9% to 86.9%, irrespective of the acid source. Although new corrosion products form in these acidic environments, the adverse effects of NH_4_/P loss from struvite, along with the redissolution of corrosion products due to their higher solubility, increase porosity and subsequently reduce the mechanical strength. Nevertheless, considering that strength retention is significantly higher than that of other cement-based materials reported in the literature, MPC still exhibits good acid resistance and is suitable for environments requiring enhanced resistance to acid corrosion.

## 1. Introduction

Cementitious materials are essential for use in environments that are both chemically and biologically aggressive, particularly those with lower pH values [1]. Biogenic sulfuric acid corrosion, prevalent in settings with elevated hydrogen sulfide (H_2_S) levels, moisture, and atmospheric oxygen, is a rapid mechanism of concrete deterioration, typically occurring within a pH range of 1.0 to 2.0 [2,3,4]. Usually, various types of calcium-based cement behave in an acid environment more or less as acidic unresistant binders. In addition to calcium-based cement concrete [5], water glass and sulfur-based concrete [6] have been developed to resist the erosion of various acids, which have especially good corrosion resistance to sulfuric acid and nitric acid. However, the durability and strength development of water glass and sulfur-based concrete are often inadequate, limiting their large-scale application. Concrete degradation always occurs in acid-resistant tanks, electroplating tanks, and other chemical industries, in which acid-resistant concrete is needed. The need for innovative acid-resistant cementitious materials is expected to increase significantly in the future, driven by the development of infrastructure and the intensifying aggressiveness of external environments affecting structural components [1].

Magnesium phosphate cement (MPC), categorized as a type of chemically bonded ceramic [7], has garnered significant interest. Its solidification is typically driven by the acid–base aqueous reactions occurring between acidic phosphates and dead-burnt MgO [8,9,10]. MPC is recognized for its exceptional properties, including rapid strength development [11], outstanding volumetric stability [12], superior fluidity [13], and a strong adhesive ability to existing substrates [14]. As a cementitious material that undergoes hydration and hardening via acid–base reactions, MPC inherently possesses a certain level of acid resistance. The major component of MPC, dead-burnt MgO, is crucial in both the hydration mechanism and the resultant hardened characteristics. For the optimal formation of MPC, a mass ratio of MgO to phosphates typically ranging from 3:1 to 5:1 is recommended [9,15], with only up to 25% of the total MgO participating actively in the hydration process [16]. This indicates that a significant amount of dead-burnt MgO remains unreacted in the hardened MPC paste. Could the presence of low-reactivity MgO significantly enhance the acid resistance of MPC, offering a novel pathway for developing acid-resistant cementitious materials?

Acidic phosphates play positive roles in the hydration process and hardening performance of MPC [13,17]. However, previous studies have indicated that the residual phosphates remaining in the hardened MPC paste negatively impact its water resistance [18,19], which is considered a primary drawback. This is demonstrated by a reduction in compressive strength and mass upon water exposure, mainly resulting from the leaching of residual phosphates and the instability of hydration products [18,20,21,22]. Additionally, as one of the main components of MPC, phosphate can resist the addition of small amounts of acid and alkali from the outside while maintaining a stabilized pH value, which is called the buffering effect [23]. Phosphates act as buffers due to their ability to exist in multiple ionic forms that can interconvert. Within a pH range of approximately 2.0 to 12.0, the equilibrium between H_2_PO_4_^−^ and HPO_4_^2−^ allows for proton exchange, stabilizing the solution’s pH. The buffering effect of phosphates is influenced by their ionic forms, yet there is limited research on how the phosphates and their buffering action in magnesium phosphate cement contribute to its acid resistance.

The acid–base reaction characteristics during the hydration and hardening process of MPC, along with the buffering effect of phosphates and the presence of a large amount of unreacted dead-burnt MgO in the hardened cement matrix, are theoretically able to positively influence its acid resistance. Ding et al. [24] observed that when MPC was immersed in a nitric acid solution for 60 days, its compressive strength initially declined but later recovered, whereas exposure to sulfuric acid did not alter its compressive strength [25]. In another study, Yang et al. [26] reported that MPC mortar maintained in sulfuric acid across various pH levels for 7 days exhibited a notable reduction in compressive strength compared to samples cured in air or water. A previous study [27] found that pure synthesized struvite partially degraded with sulfuric acid corrosion, leading to the formation of a new crystalline hydrate, Mg_3_(PO_4_)_2_ 22H_2_O. Shi et al. [28] investigated the effects of sulfuric acid (pH = 2.0–7.0) on the hydration and hardening of MPC. While increased hydration and reduced amorphization were observed, the depletion of NH4/K, morphological changes, and increased porosity offset these benefits, ultimately reducing the strength of the material. The existing research tends to focus on studying the acid resistance of MPC in specific acidic environments, lacking comprehensive investigations into its performance in typical inorganic acid conditions. Additionally, previous experiments predominantly involved short-term corrosion testing, whereas long-term testing is often more convincing, yet research in this area remains unexplored.

The objective of this study is to evaluate the acid resistance of MPC under various acidic conditions. The long-term hydration and hardening behavior of MPC was compared across different inorganic acidic conditions with varying initial pH levels. The alterations in pH, compressive strength, phase composition, and microstructural properties were thoroughly examined. Phase composition and microstructure were characterized using X-ray diffraction (XRD), thermogravimetric analysis (TG), mercury intrusion porosimetry (MIP), and scanning electron microscopy (SEM). The findings provide a theoretical foundation for the engineering applications of MPC and offer new insights into the effects of acidic environments on the phase transformation, long-term stability, and durability of MPC.

## 2. Materials and Methods

### 2.1. Materials and Sample Preparation

**Figure 1:**
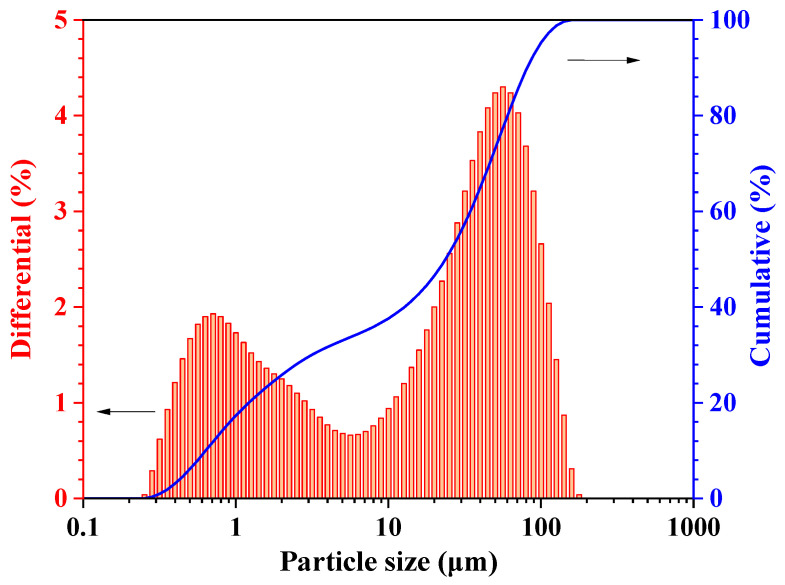
The particle size distribution of dead burnt MgO.

**Figure 2:**
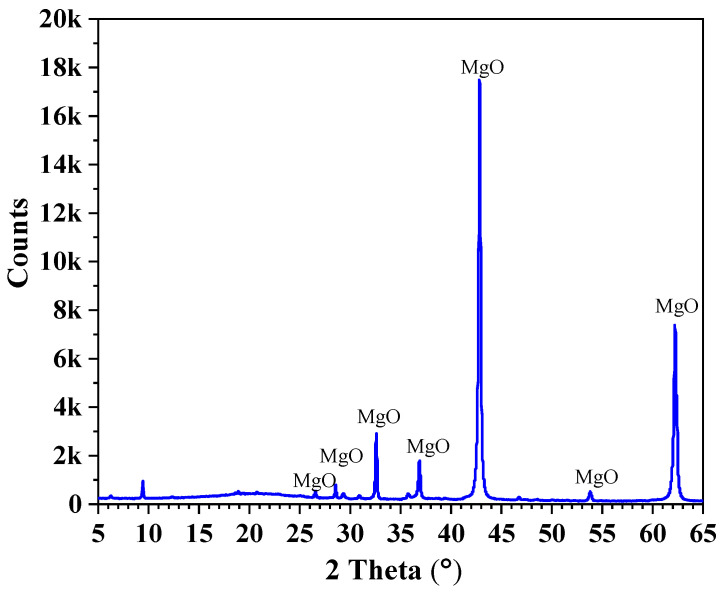
The XRD pattern of dead burnt MgO.

Commercially produced dead-burnt MgO was sourced from Haicheng Magnesite Group Co., Ltd., Haicheng, Liaoning Province, China. Technical-grade NH_4_H_2_PO_4_ and borax, each with a purity greater than 99%, were sourced from Chongqing Sanjiang Chemicals Co., Ltd., Jiaxing, China. The granular NH_4_H_2_PO_4_ was ground to finer powders with required particle size and then passed through a 100-mesh square sieve. The particle size distribution and XRD analysis of the dead-burnt MgO powders are illustrated in Figure 1 and Figure 2, respectively. The chemical composition of dead burnt MgO is detailed in Table 1.

MgO (M), NH_4_H_2_PO_4_ (P), and borax (B) were utilized to prepare MPC cement. The mass ratio of MgO to NH_4_H_2_PO_4_ (M/P) was maintained at 3:1. The mass ratios of water to cement (W/C) and borax to MgO (B/M) were kept constant at 0.12 and 0.10, respectively, as shown in Table 2. The determination of M/P, W/C, and B/M was based on the reported superior mechanical performance of MPC [29,30]. The MgO powder, NH_4_H_2_PO_4_, and borax were initially mixed at a low speed of 300 rpm for 30 s, followed by high-speed mixing at 1000 rpm for an additional 3 min. The freshly prepared cement paste was poured into molds measuring 25 mm × 25 mm × 25 mm. After 6 h of curing, the cement specimens were demolded and stored in ambient conditions (21 ± 1 °C and 65 ± 5% relative humidity) for 3 days before being exposed to inorganic acid solutions.

**Figure 3:**
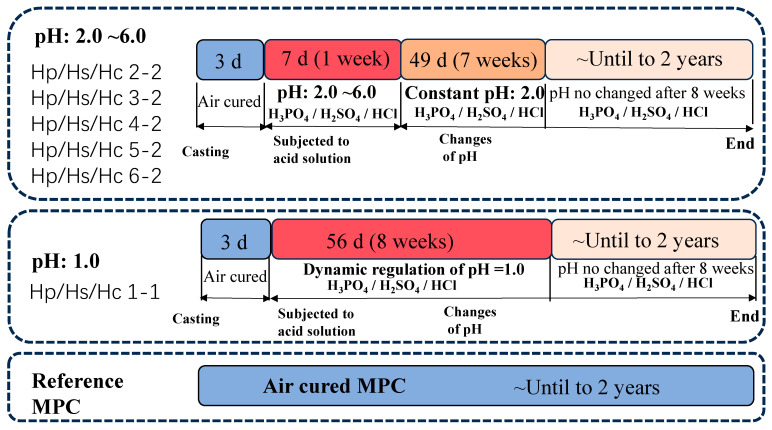
The acid attack regimes for MPC.

In this study, three inorganic acids—HCl, H_2_SO_4_, and H_3_PO_4_—were selected as the acid sources. The pH values of these acidic solutions were adjusted to 1.0, 2.0, 3.0, 4.0, 5.0, and 6.0, reflecting the pH range commonly found in degraded sewer pipes due to biological processes and acid rain. Considering that corrosive environments typically exhibited fluctuating rather than constant pH levels, the cement specimens were initially exposed to environments with varying pH conditions, followed by immersion in a stable pH environment to more accurately simulate real-world sulfuric acid corrosion. The variable pH of the three acid solutions ranged from 2.0 to 6.0 during the first 7 days (1 week), after which the pH was maintained at a constant value of 2.0 during for another 49 days (7 weeks), and then it was not changed until for 2 years. In addition, another batch of specimens was exposed to inorganic acids with the dynamic regulation of pH at 1.0 for the first 8 weeks, after which it was continually exposed to acids with pH unchanged for 2 years of immersion. The reference sample was cured in the air for two years with ambient conditions of 21 ± 1 °C and 65 ± 5% relative humidity. The detailed curing and acid corrosion regimes for MPC are shown in Figure 3. The sample nomination is based on the type of corrosive medium and its pH adjustment over time. For instance, “Hp 3-2” designates a sample exposed to phosphoric acid (H_3_PO_4_) with an initial pH of 3.0, which was subsequently adjusted to pH 2.0. Additionally, “Hs” represents exposure to H_2_SO_4_, while “Hc” indicates exposure to HCl.

### 2.2. Experimental Methods

#### 2.2.1. Compressive Strength, Mass Loss, and pH Changes

The compressive strength of the cement samples was evaluated at different curing ages based on the Chinese national standard GB/T 17671:1999 [31], in accordance with ASTM C109/C109M [32]. The loading rate during testing was set at 1.2 kN/s. The average values from six separate measurements were used as the representative compressive strength. The compressive strength retention ratio of MPC after acid erosion was calculated. The strength retention ratio, M_t_, was determined using Equation (1), where f_t_ represents the compressive strength of MPC pastes submerged in acid solutions for 2 years, and F_t_ indicates the compressive strength of air-cured MPC pastes over the same period [33].

M_t_ = f_t_/F_t_ (1)

To evaluate mass loss before and after acid exposure, the MPC specimens were removed from the corrosive solution, and their surface moisture was gently wiped using absorbent paper to maintain a saturated surface-dry condition. The mass of the surface-dried MPC specimen (m_2_) was then measured and compared to its pre-exposure mass (m_1_). The mass loss rate was subsequently calculated as the difference between these two measurements, allowing for a quantitative assessment of degradation [34].

The variation in ion concentration within the corrosive solution could reflect the chemical interactions between magnesium phosphate cement (MPC) and inorganic acids. The final pH value of the corrosive solution after 2 years of MPC exposure was tested using a pH meter (FE 28), manufacturered by Metler Toledo Technology (China) Co., Ltd., Shanghai, China.

#### 2.2.2. Mineralogical Analysis Methods

After 2 years, the air-cured and acid-attacked hardened MPC pastes were crushed into small segments with sizes of 1~2 cm. For further microscopic characterization, the cement segments were soaked in isopropanol for 3 days and then dried at 40 ± 0.5 °C for 3 days to remove the free water and the remaining isopropanol. One batch of the dried segments was ground to powders passing 75 μm sieves for further XRD and TG-DTG analysis. The other batch of the dried segments was subjected to SEM and MIP analysis.

X-ray diffraction (XRD) analysis was performed to identify the crystalline phases modified by acid exposure. The diffraction patterns were obtained using a Malvern Panalytical (Almelo, The Netherlands) diffractometer (Empyrean) equipped with Cu-Kα1 radiation (λ = 1.54 Å, 45 kV, 45 mA) and utilizing the backloading technique. Data were collected over a 2θ range of 5–65°, with a scanning speed of 2° (2θ) per minute and a step size of 0.02° (2θ).

Thermogravimetric (TG) and derivative thermogravimetric (DTG) analyses were performed using a Netzsch (Bavaria, Germany) STA449F3 apparatus. The temperature was increased from 20 °C to 1000 °C at a heating rate of 5 °C per minute under a nitrogen atmosphere to prevent oxidation. Approximately 20~30 mg of the cement powers was placed in an alumina crucible, with a nitrogen flow rate of 50 mL/min maintained during TG-DTG tests.

#### 2.2.3. Microstructural Analysis

The morphology of freshly fractured surfaces and the chemical composition of hydrates or corrosion products in hardened MPC pastes were examined using a Quattro S scanning electron microscope (SEM) from Thermo Fisher Scientific (Waltham, MA, USA), equipped with energy-dispersive spectroscopy (EDS). SEM images were captured at an accelerating voltage of 15 kV.

The pore characteristics of the hardened MPC pastes subject to acid attack were determined using a mercury intrusion porosimeter (MIP, MicroActive AutoPore V 9500) from Micromeritics (Norcross, GA, USA). The contact angle and surface tension of the mercury were set at 130° and 0.48 N/m, respectively.

## 3. Results and Discussion

### 3.1. Compressive Strength and Mass Loss

**Figure 4:**
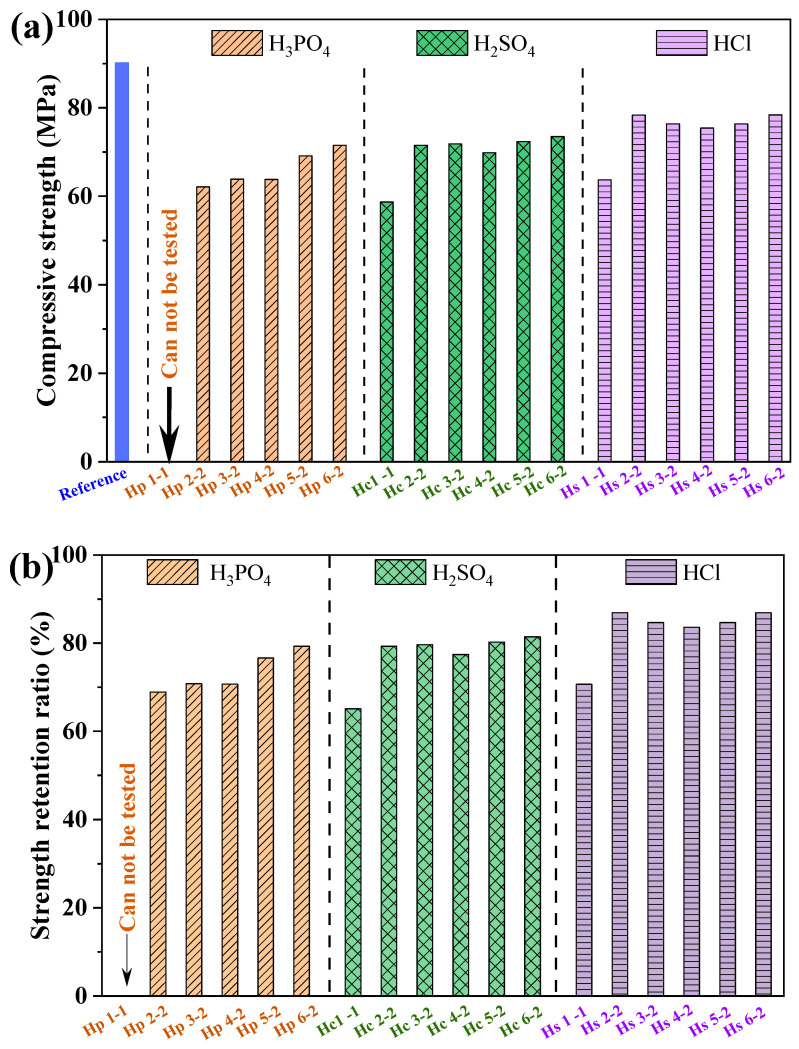
The (**a**) compressive strength and (**b**) strength retention ratio of MPC paste subjected to different acid solutions with various initial pH at two years immersion.

The influence of different inorganic acids on the compressive strength and the corresponding retention ratio of MPC paste with two years of immersion in acid solution is illustrated in Figure 4. The reference sample, which was not exposed to acid corrosion, exhibited the highest compressive strength of 90.19 MPa. For samples exposed to H_3_PO_4_, a reduction in strength was observed, particularly at lower pH levels. In contrast, exposure to H_2_SO_4_ resulted in a relatively stable compressive strength, while HCl-exposed samples maintained higher strength across all tested pH values. It can be seen that the compressive strength retention ratio increased with the rise in pH, regardless of the source of the inorganic acid. For MPC paste subjected to H_3_PO_4_ acid environments, compressive strength retention varied in the range from 68.9% to 79.3%, indicating moderate degradation. In addition, the compressive strength of MPC subjected to H_3_PO_4_ erosion with a pH of 1.0 could not be measured due to the intensive acid corrosion reaction, leading to missing edges and corners. MPC subjected to H_2_SO_4_ corrosion demonstrated relatively higher strength retention, with retention ratios between 65.1% and 81.4%. MPC subjected to HCl erosion showed the best results, with strength retention in the range of 70.6% to 86.9%, indicating excellent durability in this acid across all pH levels.

It was noteworthy that MPC specimens exposed to HCl exhibited the best acid resistance. MPC specimens exposed to sulfuric acid showed moderate strength retention. Phosphoric acid immersion resulted in the least strength retention across all pH levels. This indicates that MPC had the highest durability in HCl environments, followed by H_2_SO_4_, and the least in H_3_PO_4_ conditions, suggesting that the type of acid and its pH level significantly influence the long-term durability of MPC. The reduction in the strength of MPC exposed to inorganic acid solutions may be attributed to the following changes in mineral composition and alterations in microstructural compactness [13,27,28]: (1) the dissolution of hydrates; (2) the accelerated transformation of other hydrates, such as amorphous struvite, into crystalline struvite under acidic conditions; (3) the formation of new corrosion products (e.g., MgHPO_4_·3H_2_O).

The composite Portland cement samples containing metakaolin additives showed a compressive strength retention rate of 53.26% to 62.08% after 56 days of H_2_SO_4_ exposure [34]. Gunjal et al. [35] investigated the acid resistance of limestone calcined clay cement compared to Portland cement, reporting compressive strength retention ratios of 60.39% and 80.16% for limestone calcined clay cement and Portland cement, respectively. Graphene-modified Portland cement retains 80.04% to 85.68% of its strength after sulfuric acid exposure, significantly higher than the 55.41% to 60.39% retention of unmodified reference Portland cement [36]. The compressive strength retention rate of Portland cement modified with calcium sulphoaluminate cement after 70 days of exposure to 0.5% H_2_SO_4_ ranges from 70.0% to 85.0%, while under 5% H_2_SO_4_ exposure, the retention ratio decreases to between 30.0% and 55.0% [37]. Generally, it was evident that MPC exhibited better long-term acid resistance, significantly outperforming that of Portland cement, whose strength retention rate reportedly dropped below 30% after a 2-year acid exposure test [1].

**Figure 5:**
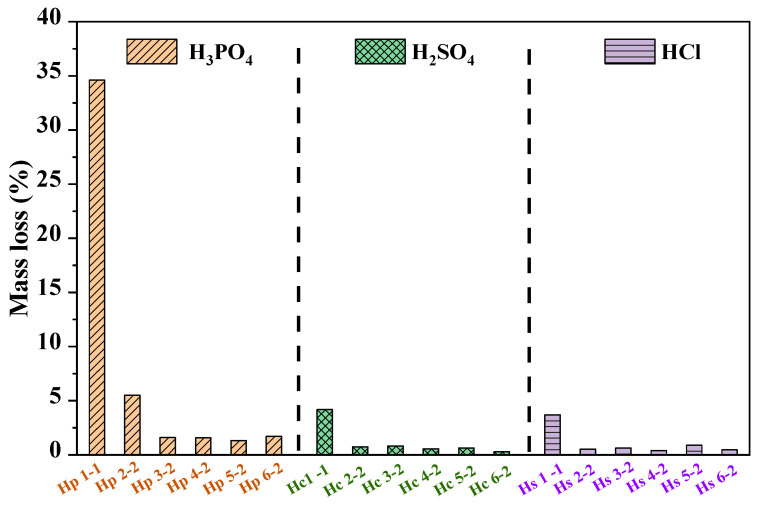
The mass loss of MPC paste subjected to different acid solutions with various initial pH at two years immersion.

Figure 5 exhibits the mass loss of MPC paste with two years of immersion in different inorganic acid solutions. It can be seen that the mass loss decreased with the rise in pH, regardless of the source of the inorganic acid. For MPC paste exposed to H_3_PO_4_ acid environments, the mass loss varied in the range from 1.32% to 34.60%, indicating moderate degradation. The mass loss of MPC cured in H_3_PO_4_ solution with pH = 1 was even up to 34.60%, due to the dissolution of hydration products. In addition, this more significant mass loss would extensively decrease the mechanical strength, as evidenced in Figure 4. MPC subjected to H_2_SO_4_ corrosion exhibited better acid resistance, with mass loss between 0.29% and 4.19%. MPC subjected to HCl showed the best results, with mass loss in the range from 0.41% to 3.70% across all pH levels. Portland concrete containing sugarcane bagasse ash exhibited mass loss ranges of 5.50% to 9.11% after exposure to 5% H_2_SO_4_ and 3.80% to 4.00% after exposure to 5% HCl [38]. Portland cement with metakaolin showed a mass loss of approximately 5.84% after exposure to 5% H_2_SO_4_ and about 5.01% after exposure to 5% HNO_3_ [34]. The sulfuric acid resistance of wood ash-containing concrete, observed under 2% and 5% H_2_SO_4_ solutions, exhibited a mass loss ranging from 6.81% to 9.70% [39]. In terms of mass loss under inorganic acid exposure, MPC demonstrated superior acid resistance compared to other cementitious materials.

### 3.2. Apparent Changes in Surfaces of Hardened MPC Paste

**Figure 6:**
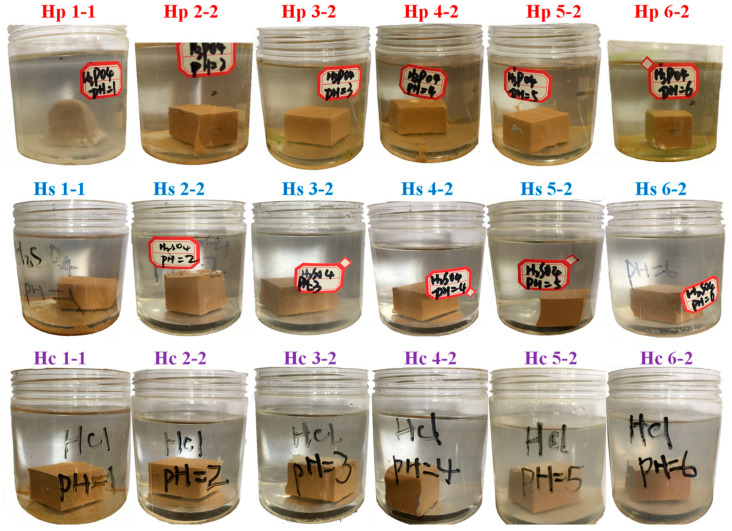
The apparent changes of MPC paste subjected to different acid solutions with various initial pH after two years immersion.

Figure 6 illustrates the appearance of MPC specimens after 2 years of immersion in different inorganic acid solutions with varying pH levels. After 2 years of exposure to different inorganic acid solutions, the MPC specimens exhibited distinct visual degradation patterns, varying according to the type of acid solution. In low pH environments, particularly in H_2_SO_4_ and H_3_PO_4_ solutions with pH values of 1.0, the specimens exhibited significant surface deterioration, with visible erosion and rounding of edges. In contrast, specimens immersed in solutions with higher pH values, especially those in the pH 2.0 to 6.0 range, exhibited less pronounced surface damage. Notably, the specimens exposed to H_3_PO_4_ solution across all pH levels displayed a less preserved external appearance compared to those in H_2_SO_4_ and HCl, likely due to different interaction mechanisms with the cement components.

### 3.3. Changes of pH

**Figure 7:**
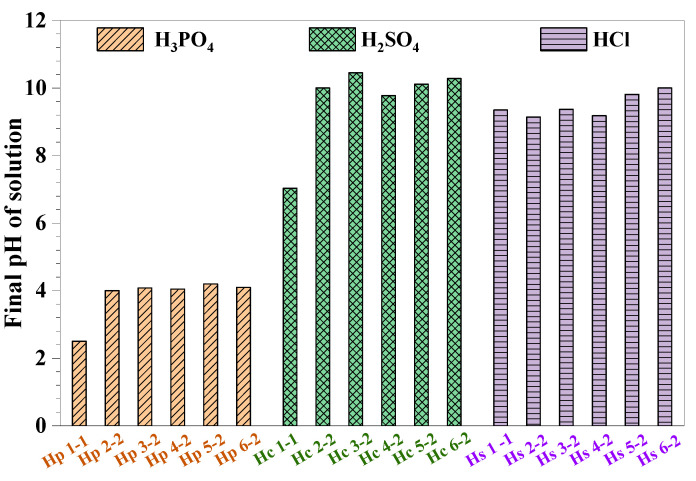
The final pH of different acid solutions with various initial pH after two years of immersion.

Figure 7 illustrates the pH variation in different inorganic acid solutions after 2 years of immersion with MPC specimens. In the H_3_PO_4_ solutions, the final pH values ranged between approximately 2.5 and 4.2, indicating moderate resistance of the MPC to phosphoric acid-induced degradation. This was attributed to H_3_PO_4_ or phosphates acting as buffers through their ability to exist in multiple interconvertible ionic forms, with the proton exchange between H_2_PO_4_^−^ and HPO_4_^2−^ effectively stabilizing the solution’s pH [23]. However, despite the relatively stable acidic conditions, the mechanical properties of the MPC, such as strength retention, showed a significant decline. This was accompanied by visible surface deterioration, including the formation of cracks and pores, suggesting that the structural integrity of the MPC was compromised despite minimal changes in pH. In contrast, the H_2_SO_4_ solutions showed a more pronounced increase in pH compared to the H_3_PO_4_ solutions, indicating a more aggressive interaction between sulfuric acid and MPC matrix. In the case of HCl, the pH rise was similar to that for H_2_SO_4_, demonstrating the strong neutralizing effect of MPC subjected to HCl environments. The excess MgO in the hardened MPC paste, due to its high Mg/P ratio, dissolves more readily in the initial H_2_SO_4_ and HCl solutions, continuously releasing OH^−^ ions, which shifts the pH of the immersion solutions toward an alkaline state.

Given the substantial reduction in both strength and specimen quality in H_3_PO_4_ environments with a pH value of 1.0, it can be concluded that MPC did not exhibit satisfactory resistance to phosphoric acid attack, despite the limited pH variation. A previous study [40] on the acid resistance of Portland cement and geopolymer binders showed that Portland cement had significantly higher acid neutralization capacity (obvious pH arise) and greater ion leaching. In contrast, the pH of geopolymer binders remained below 3.5, indicating lower leaching and reduced acid resistance.

### 3.4. Mineralogical Evolutions of MPC Paste

#### 3.4.1. XRD Analysis

**Figure 8:**
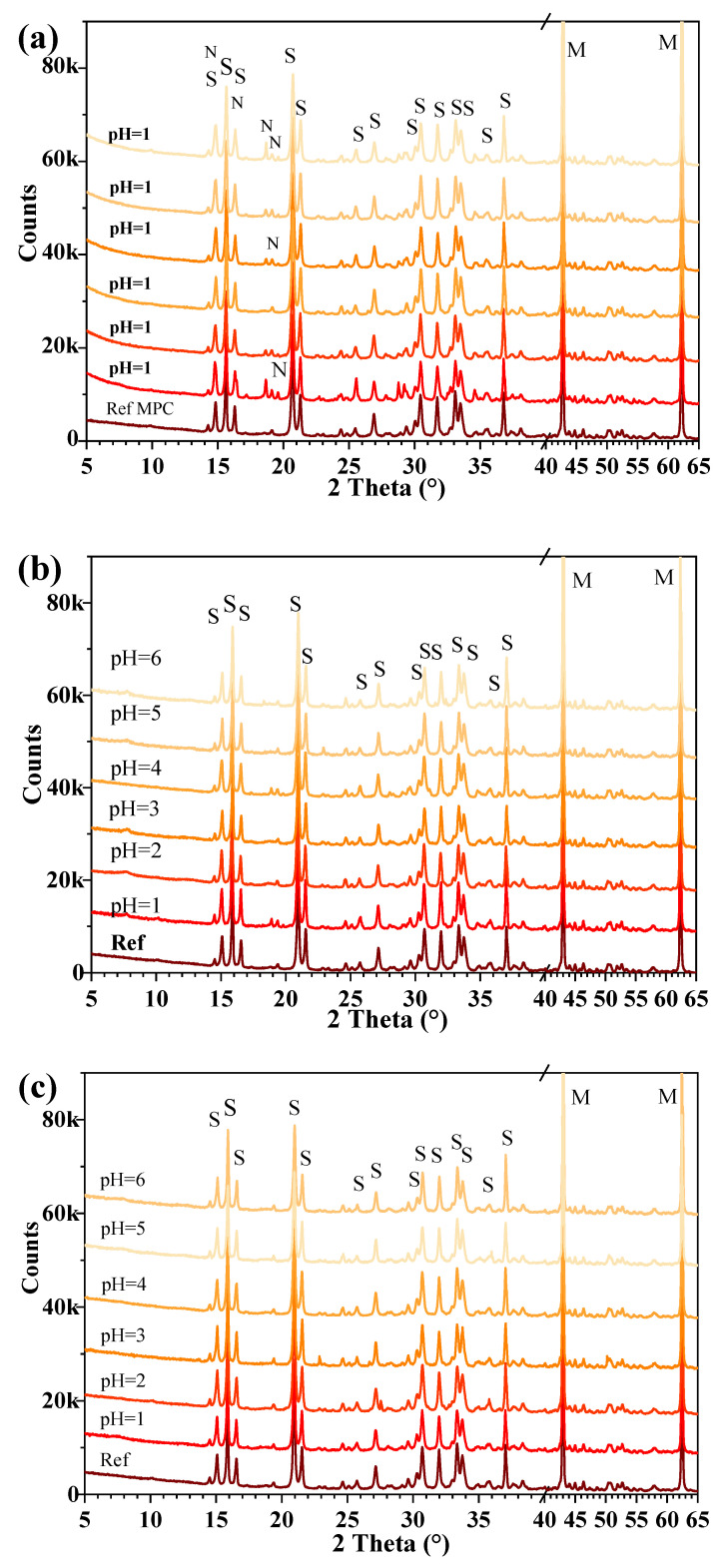
The X-ray diffraction patterns of hardened MPC pasted exposed to different inorganic acids with varying pH levels: (**a**) exposed to H_3_PO_4_, (**b**) exposed to H_2_SO_4_, (**c**) exposed to HCl. M: periclase (MgO), S: struvite (MgNH_4_PO_4_·6H_2_O), N: newberyite (MgHPO_4_·3H_2_O).

Figure 8 exhibits the XRD patterns of MPC samples subjected to different inorganic acids with varying pH. In the air-cured MPC reference, the main phases present in hardened cement paste were struvite and unreacted MgO. Figure 8a shows that, in addition to the primary hydration product struvite, newberyite (MgHPO_4_·3H_2_O) was also identified in MPC exposed to H_3_PO_4_ corrosion [22]. In MPC exposed to H_3_PO_4_, the diffraction peaks of struvite intensified progressively with increasing pH, indicating the reduced stability of struvite in highly acidic environments [28].

Figure 8b illustrates the XRD results for hardened MPC paste exposed to H_2_SO_4_ solutions across different pH levels. No new crystalline phases were detected in sulfuric acid-treated MPC samples, while struvite showed lower stability in environments with higher acidity. Similarly, Figure 8c reveals that no new crystalline phases formed when hardened MPC pastes were immersed in HCl, with HCl exposure showing negligible impact on struvite crystallization. However, intensive interactions between MgO and HCl forming soluble MgCl_2_ still occurred, though MgCl_2_ was not detected by the XRD methods due to its higher solubility in solution.

Table 3 illustrates the Gibbs free energy change (ΔG°) and solubility product constant (Ksp) for MgO reactions with different inorganic acids [41,42,43]. It reveals that the interactions between MgO and the three inorganic acids could occur spontaneously, as evidenced by the negative ΔG°. The reaction of MgO with H_3_PO_4_ yielded MgHPO_4_·3H_2_O, exhibiting a very low Ksp of 1 × 10^−24^, indicating the formation of highly insoluble precipitation. It is expected to show better resistance to phosphoric acid by MPC due to the formation of highly insoluble MgHPO_4_·3H_2_O, which could protect the cement matrix. In contrast, the formation of soluble products of MgSO_4_ and MgCl_2_ might indicate the poor resistance of MPC exposed to H_2_SO_4_ and HCl. However, the acid resistance of MPC was also influenced by other factors, such as the binding capacity of struvite, the pore structure, and the compactness of the microstructure in hardened cement paste.

#### 3.4.2. TG-DTG Analysis

**Figure 9:**
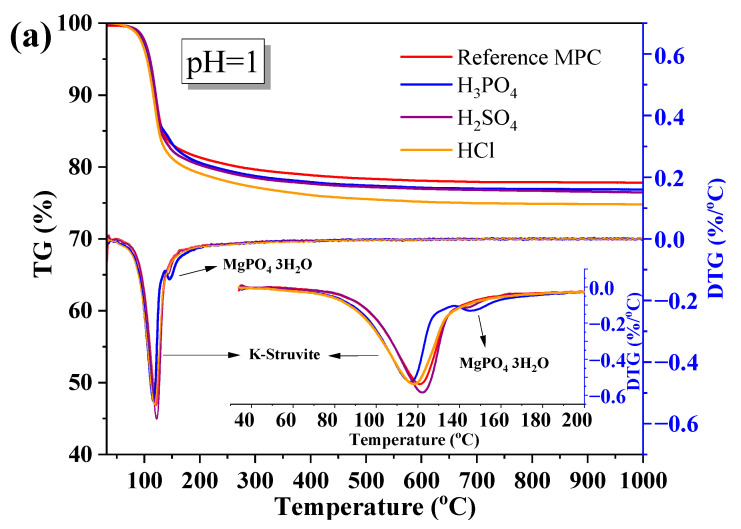

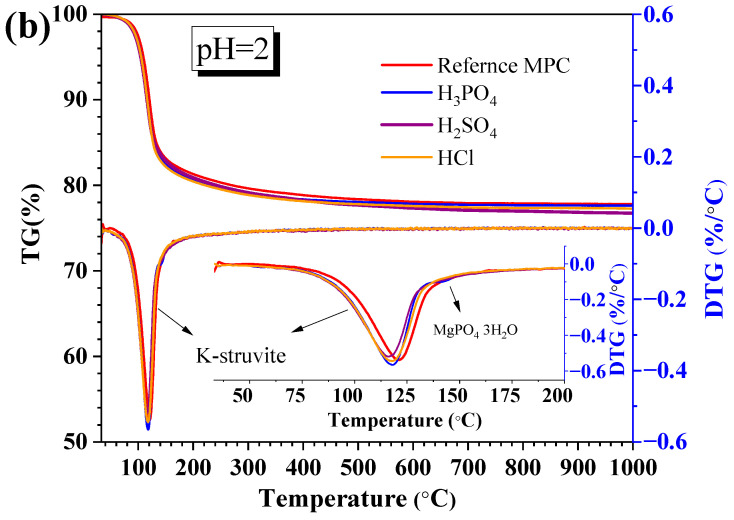
TG-DTG patterns of hardened MPC pasted exposed to different inorganic acids with varying pH levels: (**a**) pH = 1, (**b**) pH = 2.

Figure 9 presents the TG-DTG patterns of hardened MPC paste subjected to different inorganic acids at pH values of 1.0 and 2.0. All samples exhibited significant mass loss between 100 and 135 °C, attributed to the dehydration of struvite. In MPC samples exposed to H_3_PO_4_ at pH = 1, an additional notable mass loss between 150 and 175 °C corresponded to the dehydration of crystalline hydrate MgHPO_4_·3H_2_O, as indicated by pronounced peaks in the DTG curves. This suggested that H_3_PO_4_-treated specimens, particularly under highly acidic conditions, informed a higher proportion of phosphatic phases due to reactions between phosphate ions and magnesium. In contrast, MPC samples exposed to H_2_SO_4_ and HCl did not exhibit the formation of any dehydrated phases besides struvite. This indicated that sulfuric acid and hydrochloric acid primarily induce the decomposition of struvite without generating additional dehydration products.

Samples immersed in weaker sulfuric acid (pH = 2.0) exhibited a slightly higher crystalline struvite content than those exposed to stronger H_2_SO_4_ (pH = 1.0), likely due to greater struvite dissolution in the more concentrated acid. In general, the highest acidity (pH = 1) led to more aggressive degradation, evidenced by higher weight loss and sharper DTG peaks. At pH = 2.0, the extent of degradation was less pronounced, indicating that MPC retains more of its structural integrity in milder acidic environments. When MPC was subjected to inorganic acid exposure with pH = 2.0, the degradation peak intensity and temperature remained similar, indicating that the degree of deterioration decreased as the pH increased. This positively influenced the development of mechanical performance, as indicated by the higher compressive strength retention ratios when the pH was above 2.0.

### 3.5. Microstructural Changes of MPC Pastes Subjected to Acid Attack

#### 3.5.1. SEM Observations

**Figure 10:**
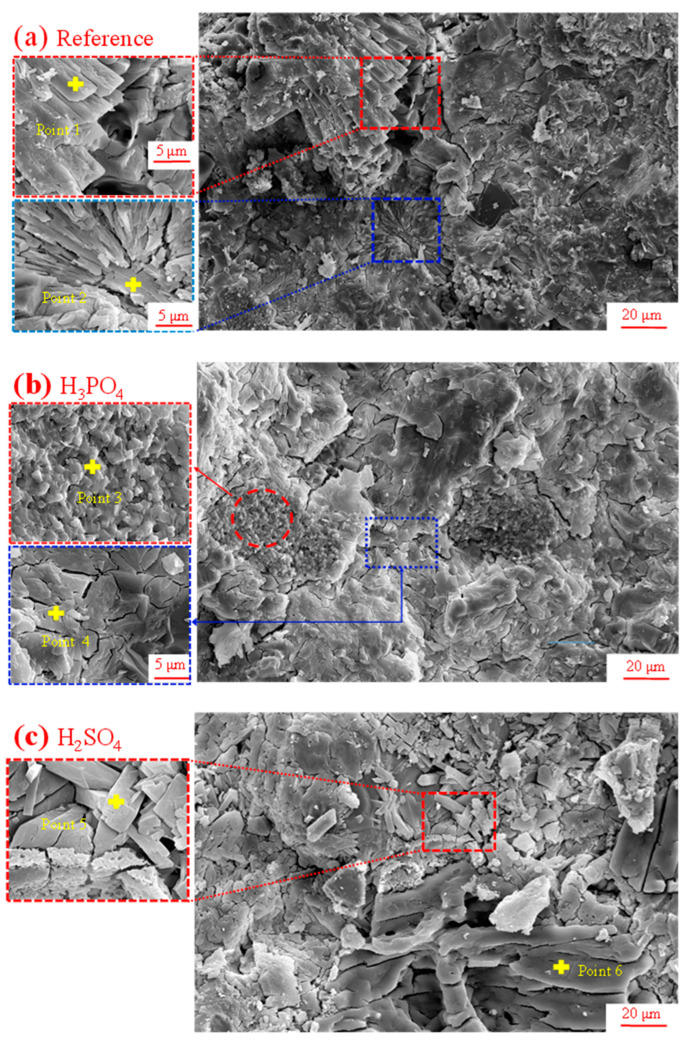

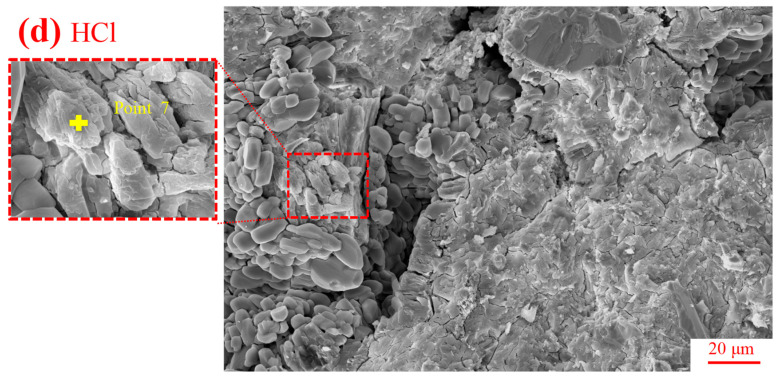
The SEM images of the hardened MPC pastes subjected to different inorganic acids with pH = 2.0 after two years.

The microstructures of the hardened MPC pastes subjected to different inorganic acids at pH = 2.0 are illustrated in Figure 10. In the reference MPC paste without inorganic acid erosion, it can be observed that the hydration products interlocked with each other to form a dense microstructure, as shown in Figure 10a. This densification was the reason why MPC typically exhibited high mechanical performance. The plate-like and tabular-like/rod-like hydrate was attributed to struvite. Figure 10b presents the microstructure of MPC after 2 years of exposure to H_3_PO_4_ solution. The newly formed hydrates with refined and granular morphologies originated from the interactions between MgO and H_3_PO_4_ forming MgHPO_4_·3H_2_O. Additionally, plate-like and fractured struvite formed in the hardened cement matrix, likely due to mechanical stress or the weakening of chemical bonds caused by acid corrosion. The EDS analysis indicated that the chemical composition of struvite deviated from its ideal stoichiometry (MgNH_4_PO_4_·6H_2_O, with a Mg/N/P molar ratio of 1:1:1) due to a higher phosphorus content, as shown in Table 4.

After two years of exposure to H_2_SO_4_ solution with pH = 2, the MPC paste exhibited distinct surface erosion morphologies and hydrate formations, as shown in Figure 10c. Large struvite crystals, characterized by their high degree of crystallinity, were observed throughout the cement matrix. The EDS analysis indicated that the chemical composition of struvite deviated from its ideal stoichiometry (MgNH_4_PO_4_·6H_2_O, with a Mg/N/P ratio of 1:1:1) due to a lower nitrogen content, which is consistent with a previous study [28].

Figure 10d shows the microstructure of MPC paste subjected to HCl erosion at pH = 2. Irregularly shaped plate-like crystals can be observed in the image, with the enlarged yellow box highlighting the detailed characteristics of these plate-like structures. In addition, loosely packed oolitic minerals were also identified, which, according to EDS results, were confirmed to be struvite. This could be attributed to the recrystallization or dissolution–precipitation process of hydration products induced by the acidic environment (HCl, low pH). The EDS analysis of these hydrates revealed a noticeable depletion of N in the struvite structure, as shown in Table 4.

#### 3.5.2. Pore Structure of MPC Pastes

**Figure 11:**
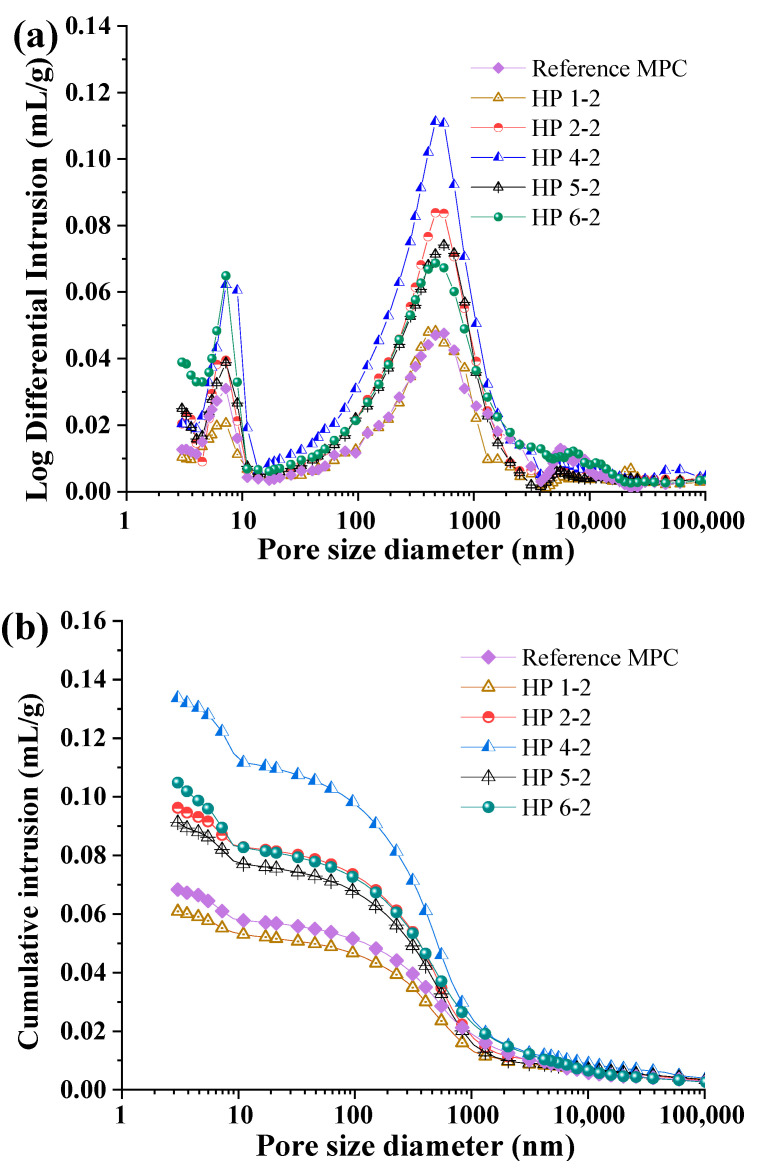
Pore size distributions of MPC subjected to H_3_PO_4_ with varying pH levels after 2 years: (**a**) log differential intrusion, (**b**) cumulative intrusion.

Figure 11 illustrates the pore structure of hardened MPC paste subjected to H_3_PO_4_ with various pH values for 2 years as determined by MIP analysis. The reference MPC paste (unexposed to acid) displayed a peak in the mesopore range, indicating a relatively stable microstructure. After being subjected to H_3_PO_4_ acid erosion for 2 years, the two main threshold pore diameters (<10 nm and 100~1000 nm, Figure 11a) exhibited a notable increase with the decrease in the pH of the environment. Furthermore, the cumulative intrusion, which corresponded to the total porosity, also apparently increased.

Usually, a severe acidic environment accelerates the dissolution of hydration products, leading to the enlargement of pore structures. Hp 2-2 showed a similar trend, but to a lesser extent, while samples Hp 4-2, Hp 5-2, and Hp 6-2 exhibited a more moderate shift, indicating that higher pH values resulted in less pronounced damage to the pore structure. In contrast, Hp 1-2 displayed a different behavior. While there was a slight increase in the pore volume for diameters below 10 nm, the overall cumulative mercury intrusion was lower compared to the reference group, likely due to the formation of MgHPO_4_·3H_2_O, which optimized the pore structure. However, the vigorous reaction between the remaining MgO in the magnesium phosphate cement and H_3_PO_4_ led to significant surface spalling and dissolution, ultimately making the samples unsuitable for strength testing.

The overall trend we observed was consistent with a previous study [44], where prolonged exposure to highly acidic environments led to the dissolution of original hydration products, particularly at lower pH levels. The formation of larger pores and the increase in total porosity were indicative of the weakening of the cement matrix, which could negatively impact mechanical properties such as compressive strength.

**Figure 12:**
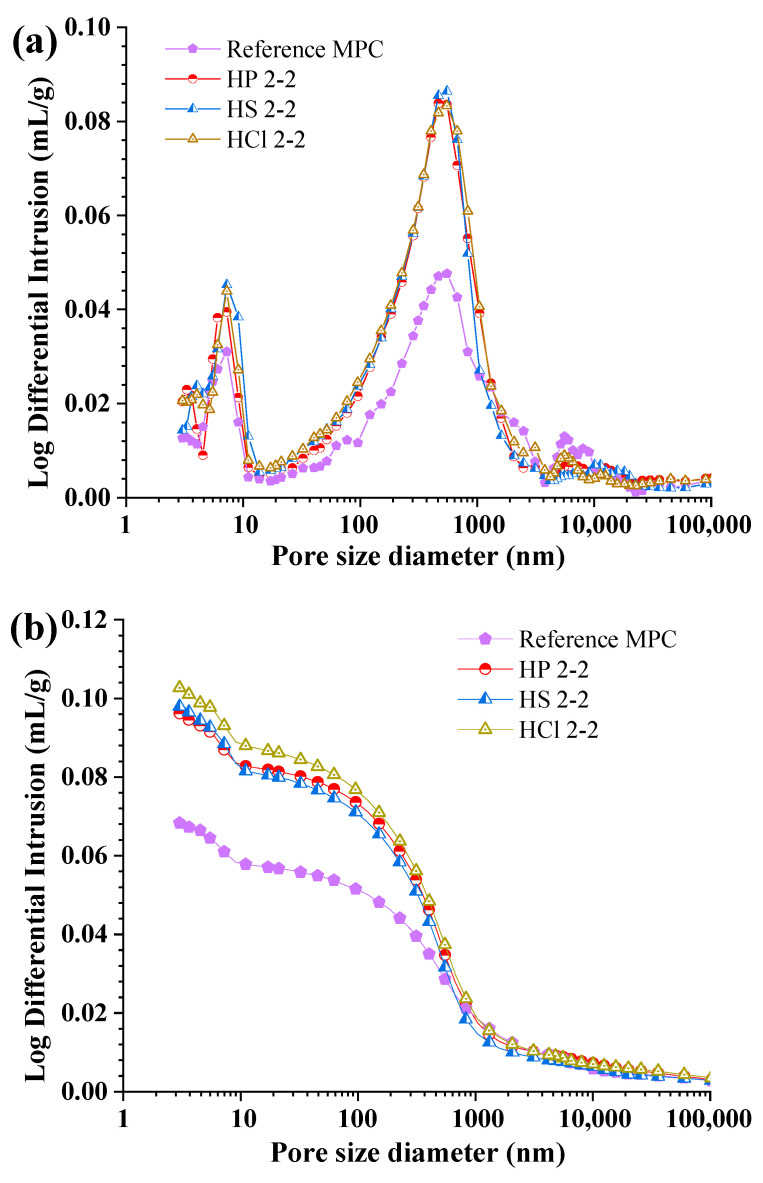
Pore size distributions of MPC subjected to different inorganic acids: (**a**) log differential intrusion, (**b**) cumulative intrusion.

Figure 12 illustrates the pore structure of hardened MPC paste subjected to different inorganic acids with a constant pH of 2.0 for 2 years, with unexposed MPC used as a reference for comparison. After being subjected to inorganic acid solutions with a pH value of 2.0 for 2 years, the two main threshold pore diameters also exhibited a notable increase, as well as the cumulative intrusion, compared with that of the reference MPC. The dissolution of original hydration products and interactions between remaining MgO and inorganic acids led to pronounced damage to the pore structures.

**Figure 13:**
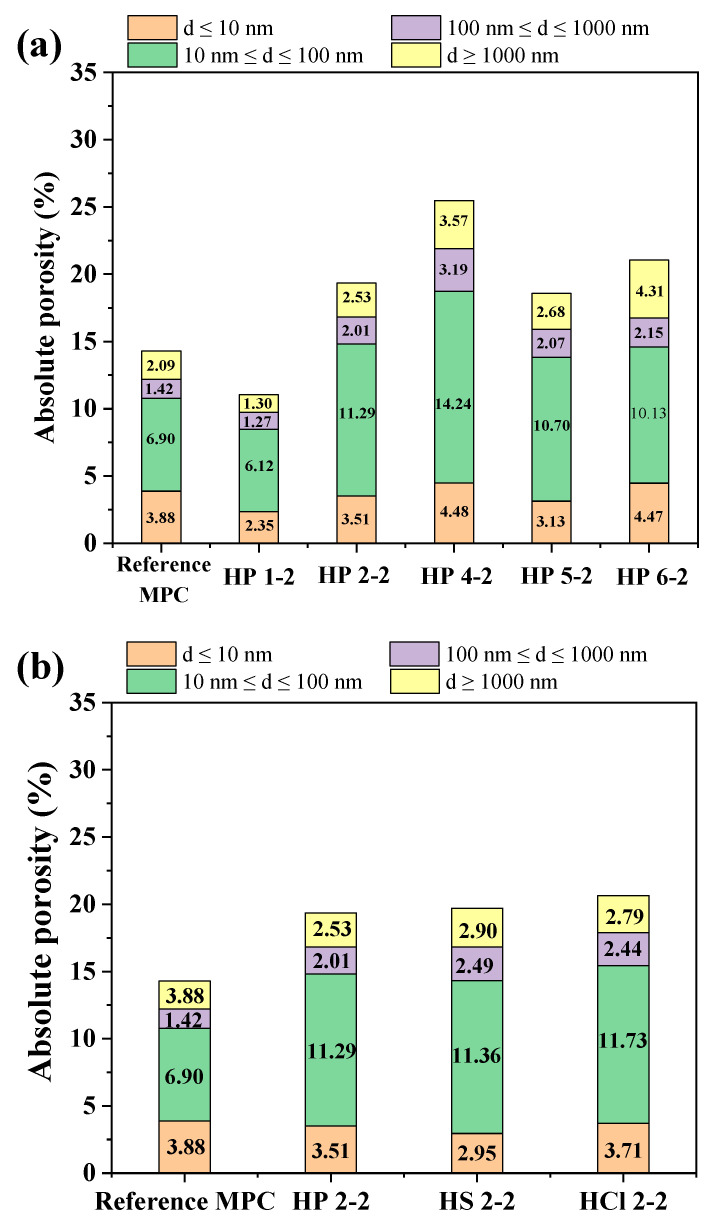
Porosity of hardened MPC produced in various curing environments: (**a**) MPC subjected to H_3_PO_4_ with varying pH levels; (**b**) MPC subjected to different inorganic acids at constant pH of 2.0.

Figure 13 illustrates the porosity of hardened MPC at various curing environments. Porosity was categorized by pore size [45]: gel pores (d ≤ 10 nm), transitional pores (10 nm ≤ d ≤ 100 nm), capillary pores (100 nm ≤ d ≤ 1000 nm), and macropores (d ≥ 1000 nm). Figure 13a displays the porosity of hardened MPC paste subjected to H_3_PO_4_ with varying pH levels. The reference MPC displayed a balanced distribution with a significant portion of gel and transitional pores, indicating a dense microstructure. Specially, for MPC subjected to H_3_PO_4_ with an initial pH value of 1.0 and subsequent pH value of 2.0, the gel pores increased slightly while transitional pores decreased, suggesting an initial refinement of the pore structure, though with a slight rise in macropores. As the pH increased from 2.0 to 6.0, the transitional pores dominated, and both capillary and macropores increased, indicating a shift towards larger pore sizes. Figure 13b displays the porosity of MPC exposed to different inorganic acids at a constant pH value of 2.0. The total porosity of MPC exposed to acid showed the following general trend: the total porosity increased, and the dominated transitional pores increased, as demonstrated by the reduction in compressive strength (Figure 4) and supported by SEM observations (Figure 10b). Notably, in MPC exposed to H_3_PO_4_, the detrimental effect of increased porosity on compressive strength surpassed the beneficial impact of MgHPO_4_·3H_2_O formation, despite its pore-filling capability. For MPC exposed to H_2_SO_4_ and HCl, the products formed from the interactions of MgO with H_2_SO_4_ or HCl exhibited relatively high solubility (Ksp), preventing the formation of precipitates. As a result, these products could not contribute to cementitious filling, increasing the total porosity and hindering the development of strength.

### 3.6. Brief Discussion

The results of this study indicate that the acid resistance of MPC varies in different inorganic acids. However, when comparing these results with similar studies on an international scale, we found that MPC performs better in acidic environments than OPC, which is consistent with the findings of study [24], demonstrating that MPC has significant advantages in specific acidic conditions. Additionally, a previous study [28] found that MPC cured in sulfuric acid with a pH higher than 2 exhibited strength comparable to or even greater than that of air-cured samples, which corroborates the acid resistance trend we observed in H_2_SO_4_.

Studies on the acid resistance of MPC are relatively scarce, and current research has not yet progressed to the modeling stage. Similar studies would contribute to a deeper understanding of the mechanisms underlying the acid resistance of MPC. A previous study [46] carried out the modeling of acid resistance for various cementitious binders, and the results show that the deterioration kinetics of calcium aluminate cement (CAC) are slower than those of other cements, attributed to the better thermodynamic stability of hydrogarnet and the pore-blocking effect of ettringite precipitation. The fundamental difference in acid resistance lies in the partial dissolution of CAC hydrates compared to the complete dissolution of CH and C-S-H in Portland cement. Under the experimental conditions of this study, long-term acid exposure demonstrated that magnesium phosphate cement (MPC) exhibited excellent acid resistance, attributed to the partial dissolution of MPC hydrates. The presence of pronounced struvite peaks even after 2 years of acid exposure supports this observation. Additionally, the presence of unreacted periclase (MgO) in MPC, which has low water and acid reactivity compared to traditional cement clinker, was evidenced by distinct MgO peaks even after 2 years of acid exposure. This phenomenon confirms the hypothesis proposed in the introduction: the presence of low-reactivity MgO can significantly enhance the acid resistance of MPC, providing a novel pathway for the development of acid-resistant cementitious materials.

This study reveals that magnesium phosphate cement (MPC) offers notable resistance to corrosion in acidic settings, which may be effectively utilized in various industrial applications. Identified uses include wastewater treatment structures, concrete sewage conduits, and the floors of facilities producing inorganic acids. MPC is characterized by its robust stability under such corrosive conditions, making it ideal for components of infrastructure exposed to acidic substances, particularly in wastewater treatment environments. In chemical manufacturing settings, MPC is advantageous as a lining for storage units, pipelines, or vessels due to its ability to withstand diverse chemical aggressors, thereby prolonging the operational life of these structures. Furthermore, the acid resistance of MPC contributes to the conservation and rehabilitation of historical architecture by providing protection against environmental contaminants and acid precipitation, thereby aiding in the preservation of cultural heritage.

## 4. Conclusions

To clarify the acid resistance of MPC under different acidic conditions, this study selected three inorganic acids with varying pH levels as corrosion media. The acid resistance of MPC was evaluated in terms of compressive strength retention, mass loss, phase composition, and microstructure, and an underlying mechanism for MPC’s acid resistance was proposed. Based on the experimental results we obtained, the following key conclusions can be drawn:

(1) The variation in the acid resistance of MPC under different inorganic acidic conditions is influenced by the pH level of the corrosive environment and the type of acid involved. Upon exposure to a pH value of 1.0, MPC exhibited greater sensitivity to H_3_PO_4_ compared to HCl and H_2_SO_4_, while at pH levels above 2.0, MPC demonstrated robust resistance to all tested corrosive media, with compressive strength retention ranging from 68.9% to 86.9%, irrespective of the acid source. These findings support the hypothesis that, as a cementitious material undergoing hydration and hardening through acid–base reactions, MPC inherently possesses a certain level of acid resistance.
(2) The excellent acid resistance of MPC under long-term acid exposure lies in the partial dissolution of MPC hydrates, as evidenced by the presence of pronounced struvite peaks in hardened cement paste. Additionally, the residual unreacted low-reactivity periclase (MgO) in the hardened paste also contributed to enhanced acid resistance, as evidenced by its presence in MPC paste even after 2 years of acid exposure. This supports the hypothesis that low-reactivity MgO significantly enhances MPC’s acid resistance, offering a promising pathway for developing acid-resistant cementitious materials.
(3) When MPC is subjected to strong acidic H_3_PO_4_ erosion, a vigorous reaction occurs between H_3_PO_4_ and unreacted MgO in the cement, resulting in the formation of a new corrosion product, MgHPO_4_·3H_2_O. This intense reaction alters the microstructure of the hardened cement matrix, increasing porosity and consequently reducing strength. Nevertheless, when the pH of the H_3_PO_4_ erosion environment is higher (pH > 2.0), MPC exhibits strong resistance to phosphate erosion, attributed to the ability of MgHPO_4_·3H_2_O to fill the pores, thus maintaining high strength retention ratios.
(4) When MPC was exposed to H_2_SO_4_ and HCl erosion, no new crystalline corrosion products were detected in the cement matrix. This was due to the soluble nature of the reaction products formed between MgO and H_2_SO_4_ or HCl, as indicated by their high solubility product (Ksp). However, the low porosity and dense microstructure of hardened MPC paste achieved good resistance to H_2_SO_4_ and HCl corrosion at higher pH values.
(5) MPC demonstrates varying levels of acid resistance in acid environments, underscoring its considerable potential for practical applications. These findings position MPC as a promising option for sustainable infrastructure, capable of reducing carbon emissions linked to traditional cement while offering superior long-term durability.

Despite the promising results, several limitations must be acknowledged. This study was limited to a narrow selection of acids (H_3_PO_4_, HCl, and H_2_SO_4_) and pH conditions, which may not fully capture the range of acidic environments that magnesium phosphate cement (MPC) could encounter in real-world applications. Additionally, the corrosion tests were performed under static conditions, whereas in practical applications, the erosion process is typically dynamic. The investigation was confined to examining the macro- and microstructural changes of MPC under static acid exposure. Lastly, further research is required to explore the effects of various additives or modifications to enhance the acid resistance of MPC. 

## Figures and Tables

**Table 1:** Chemical composition of dead-burnt MgO determined using XRF.

MgO	SiO_2_	CaO	Fe_2_O_3_	Al_2_O_3_	SO_3_	K_2_O	P_2_O_5_	Others	LOI
93.69	2.47	1.39	0.51	0.43	0.16	0.13	0.05	0.49	0.68

**Table 2:** The mix design and compressive strength of MPC cured in air.

M/P	W/C	B/M	Compressive Strength Cured in Air (MPa)			
			1 d	3 d	28 d	2 Year
3.0	0.12	0.10	60.35	75.29	85.77	90.19

**Table 3:** The ΔG° and Ksp for MgO reactions with different inorganic acids.

Related Chemical Reactions	ΔG°	Ksp
MgO + H_2_SO_4_ → MgSO_4_ + H_2_O	−122.33 kJ/mol	None
MgO + 2HCl → MgCl_2_ + H_2_O	−73.33 kJ/mol	None
MgO + H_3_PO_4_ + 2H_2_O → MgHPO_4_·3H_2_O	−137.73 kJ/mol	1 × 10^−30^

**Table 4:** The atomic percentage of certain points determined by EDS analysis.

Nomination	N	O	Mg	P	Na
Point 1	12.56	59.18	14.25	14.01	-
Point 2	12.71	59.36	14.67	13.26	-
Point 3	-	76.73	12.37	10.90	-
Point 4	13.02	57.51	13.84	15.63	-
Point 5	8.23	63.41	15.23	14.16	-
Point 6	5.37	64.38	3.76	6.04	20.45
Point 7	9.67	62.67	13.91	13.75	-

## Data Availability

The original contributions presented in the study are included in the article, further inquiries can be directed to the corresponding author.
